# Transcriptome Profile Analysis of Mammary Gland Tissue from Two Breeds of Lactating Sheep

**DOI:** 10.3390/genes10100781

**Published:** 2019-10-08

**Authors:** Zhiyun Hao, Huitong Zhou, Jon G.H. Hickford, Hua Gong, Jiqing Wang, Jiang Hu, Xiu Liu, Shaobin Li, Mengli Zhao, Yuzhu Luo

**Affiliations:** 1 Gansu Key Laboratory of Herbivorous Animal Biotechnology, Faculty of Animal Science and Technology, Gansu Agricultural University, Lanzhou 730070, China; haozy2018@163.com (Z.H.); huitong.zhou@lincoln.ac.nz (H.Z.); hua.gong@lincoln.ac.nz (H.G.); huj@gsau.edu.cn (J.H.); liux@gsau.edu.cn (X.L.); lisb@gsau.edu.cn (S.L.); 18394187234@163.com (M.Z.); 2 Gene-Marker Laboratory, Faculty of Agriculture and Life Science, Lincoln University, Lincoln 7647, New Zealand

**Keywords:** RNA-Seq, mammary gland, differentially expressed gene, sheep

## Abstract

The mammary gland is a crucial tissue for milk synthesis and plays a critical role in the feeding and growth of mammalian offspring. The aim of this study was to use RNA-sequencing (RNA-Seq) technology to provide a transcriptome profile of the ovine mammary gland at the peak of lactation. Small-Tailed Han (STH) sheep (*n* = 9) and Gansu Alpine Merino (GAM) sheep (*n* = 9), breeds with phenotypic differences in milk production traits, were selected for the RNA-Seq analysis. This revealed 74 genes that were more highly expressed in the STHs than in the GAMs. Similarly, 143 genes that were expressed at lower levels in the STHs than in the GAMs, were identified. Gene ontogeny (GO) and Kyoto Encyclopedia of Genes and Genomes (KEGG) enrichment analyses revealed that these differentially expressed genes (DEGs) were associated with binding and catalytic activities, hematopoietic cell lineages, oxytocin signaling pathway and neuroactive ligand–receptor interaction. This is the first study of the transcriptome profile of the ovine mammary gland in these Chinese breeds at peak lactation. The results provide for a better understanding of the genetic mechanisms involved in ovine lactation.

## 1. Introduction

The efficiency of sheep farming systems, including the breeding for replacement ewes and the production of lambs for fattening and slaughter, is principally affected by the number of lambs that survive to weaning. For this reason, sheep fertility and lamb survival are major concerns for farmers. Among the many factors affecting these traits, ewe lactation traits are of interest, especially when multiple lambs need to be raised. In this context, understanding the function of the mammary gland is of importance to improving lamb production.

If the supply of milk is insufficient, a lamb will not get enough nutrition in early life, causing slow growth and even death. With improvements in sheep nutrition in China, ewe milk yield has been greatly improved, but seasonal climatic variation means obtaining adequate nutrition year-round to enable good milk yield, may not always be possible. For example, the quality and type of forage can vary greatly, with typically more forage being available in summer and autumn. Accordingly, breeding approaches to improve lactation performance need to be considered too.

In this respect, while traditional selection approaches to improve milk yield do work, the trait has been described as having low heritability in Sarda dairy sheep [1]. Breeding using marker-assisted selection might therefore improve the accuracy of selection for milk traits, but to enable this, the challenge is to identify genes underpinning milk traits.

Next-generation sequencing (NGS) technologies have provided a unique opportunity to quantify and annotate the transcriptome of many organisms [2]. Of these NGS technologies, RNA Sequencing (RNA-Seq) enables the generation of transcriptome information. Compared to microarray, RNA-Seq is considered to have greater accuracy and sequencing depth [2].

RNA-Seq has been widely used for the identification and characterization of differentially expressed genes (DEGs), and recently it has been used in the analysis of the mammary gland transcriptome of several species including humans [3,4], cattle [5,6], yak [7], and sheep [8,9,10]. 

The yield of milk during lactation is affected by many factors including breed, nutrition and feed management, age, season, health, parity, milking frequency and environment. Some of these factors can also affect milk composition [11]. In China, the indigenous Small-Tailed Han (STH) sheep have good milk production performance, with an average milk yield at 30 days postpartum of 1130 g/d [12]. In contrast, the milk yield of the Gansu Alpine Merino (GAM) sheep is usually two-thirds of that of STH sheep. The STH sheep is indigenous to China, and is characterized by its hardiness. It is well adapted to the challenging regions in which it is farmed. The GAM sheep is a breed that has been created as a cross between the Xinjiang Merino, Caucasian Merino and other Mongolian sheep [13]. It is used for both meat and wool production and is adapted to cold or alpine environments. Both breeds are of economic importance in the localities in which they are found.

In this study, we used RNA-Seq to examine gene expression in mammary gland tissue from STH sheep and GAM sheep, with both breeds being at the peak of their lactation. An increased understanding of the DEGs in these breeds, and their participation in the key biological processes of lactation, could facilitate the development of improved approaches to increasing the milk yield of ewes.

## 2. Materials and Methods

### 2.1. Ethics Statement

Experimentation on the sheep was conducted according to the guidelines for the care and use of experimental animals, as established by the Ministry of Science and Technology of the People’s Republic of China (Approval number 2006-398). It was also approved by the Animal Care Committee of Gansu Agricultural University, Lanzhou, China.

### 2.2. Experimental Animals and RNA Preparation

The STH and GAM sheep were raised under the same environmental conditions with natural light and free access to food and water. They were extensively grazed on natural pasture at the Songshan, Tian Zhu County Sheep Farm (Gansu, China). During lactation, each ewe received an additional 300 g of cornmeal per day. Nine healthy, fourth parity, three-year-old STH ewes and nine healthy, fourth parity, three-year-old GAM ewes, were slaughtered at the peak of lactation (20 days after lambing). The average milk yield within 30 days postpartum of GAM and STH investigated in the study were 853 and 1357 g/d, respectively. The STH milk also has a higher fat (7.42% vs. 5.66%) and protein (5.37% vs. 4.67%) content than GAM milk.

The parenchyma of the mammary gland tissue was collected from individual ewes, as this contains the lobules that synthesize and secrete milk. The samples were immediately frozen in liquid nitrogen, then transferred to the laboratory and stored at −80 °C until needed.

Total RNA from the parenchyma was extracted using the Trizol Reagent (Life Technologies, USA) according to the manufacturer’s instructions. The concentration and quality of the RNA was determined using the NanoDrop 2000 (Thermo Scientific, MA, USA) and the Agilent 2100 (Agilent, CA, USA) instruments. To reduce the effect of variation between individual ewes, an equal volume of RNA from each of three ewes, randomly chosen from the nine experimental ewes of each breed, was mixed to produce six different pooled RNA samples of three ewes each (GAM-1, GAM-2, GAM-3, STH-1, STH-2 and STH-3) using a similar approach to that described by Paten et al. [14].

### 2.3. cDNA Library Construction and RNA-Seq 

Libraries (cDNA) of high quality RNA samples (RIN (RNA Integrity Number) > 7) were prepared using the Illumina® TruSeqTM RNA Sample Preparation kit (Cat. No. 15008136 A, Illumina Inc. CA, USA) following the manufacturer’s instructions. The mRNA was purified from the total RNA using poly-T oligo-attached magnetic beads (Cat. No. MRZ11124C, Illumina Inc. CA, USA). The mRNA was fragmented into 200–300 bp pieces using divalent cations at 94 °C for 6 minutes. The first strand cDNA was synthesized using random oligonucleotide primers and SuperScriptTM II reverse transcriptase (Cat. No. 18064014, Invitrogen, CA, USA), while the second strand cDNA synthesis was undertaken using DNA polymerase I. This was followed by an RNase H treatment. The remaining DNA overhangs were converted into blunt ends via exonuclease polymerase activities and then the enzymes were removed. After adenylation of the 3′-ends of the DNA fragments, Illumina PE adapter oligonucleotides were ligated to prepare for hybridization.

To select cDNA fragments of the preferred ~200 bp in length, the library fragments were purified using the AMPure XP system (Cat. No. A63880, Beckman Coulter, CA, USA). DNA fragments with ligated adaptor molecules on both ends were selectively enriched using an Illumina PCR primer cocktail in a 15-cycle PCR reaction. The products were purified (AMPure XP system, Cat. No. A63880) and quantified using the Agilent high sensitivity DNA assay on a Bioanalyzer 2100 system (Agilent, CA, USA). The chain-specific library was then subjected to paired-end sequencing by the Shanghai Personal Biotechnology Co. Ltd (Shanghai, China), using the HiSeq 2500 platform (Illumina), to yield 2× 150 bp reads.

### 2.4. Mapping of Sequencing Reads and Identification of DEGs

The quality of reads was evaluated using the FastQC (http://www.bioinformatics.babraham.ac.uk/projects/fastqc/). Clean reads were obtained by removing the raw reads to reads containing adapters and the low quality reads with quality scores < Q20 using Cutadapt Version 1.2.1 [15]. The remaining clean reads were mapped against the ovine genome assembly v.3.1 (Oar_v3.1) using TopHat v2.0.1.2 [16]. Only the unique and mapped reads were used for the gene expression analysis.

DESeq v1.18.0 [17] was used to compare gene expression levels within the same sample. Gene enrichment was normalized by library and gene length through calculating Reads per Kilo bases per Million reads (RPKM) using the Ensembl gene annotation genes (Oar_v3.1) as a reference [18]. Genes with RPKM > 0.01 in at least two samples from each library were considered to be expressed. The DESeq method was used to identify DEGs with the |log2fold change| > 1 and corrected *p*-values < 0.05 [17,19].

### 2.5. GO and KEGG Pathway Analyses 

Gene ontology (GO) analysis with the GOseq R package (http://kobas.cbi.pku.edu.cn), was used to analyze the main functions of the DEGs. The KOBAS 2.0 software (http://bioinf.wehi.edu.au/software/goseq/) was used to compare the DEGs with the Kyoto Encyclopedia of Genes and Genomes pathway database [20].

### 2.6. Validation of DEGs by Reverse Transcription-Quantitative PCR

To validate the repeatability and reproducibility of the gene expression data obtained by the RNA-Seq approach, reverse transcription-quantitative PCR (RT-qPCR) was carried out on 13 randomly selected DEGs using the individual RNA samples extracted originally for RNA-Seq. The identity of these DEGs and primer information are listed in Table 1.

The cDNA was synthesized using SuperScriptTM II reverse transcriptase (Cat. No. 18064014, Invitrogen, CA, USA). RT-qPCR was carried out in triplicate with the 2×ChamQ SYBR qPCR Master Mix (Cat. No. Q311, Vazyme, Nanjing, CHN) on an Applied Biosystems QuantStudio® 6 Flex (Thermo Lifetech, USA). The thermal profile included an initial denaturation of 30 s at 95 °C; followed by 45 cycles of 95 °C for 10 s, 60 °C for 34 s, and 95 °C for 15 s; and finished by 60 °C for 60 s. The mRNA levels of the DEGs were compared to the expression of the *β-actin* and *GAPDH* gene (house-keeping genes) in the corresponding samples [21]. The relative gene expression levels were calculated using the 2^-ΔΔCt^ method.

## 3. Results

### 3.1. RNA-Seq Reads and Mapping to the Reference Genome

Upon RNA-Seq analysis, raw reads were obtained for each library and between 136 and 141 million clean reads were obtained after quality control procedures and were applied to the STH and GAM libraries (GAM-1, GAM-2, GAM-3, STH-1, STH-2 and STH-3) (Table 2). The reads of Q30 (Quality score > 99.9%) were over 90% for all six groups, and the correlations between the three pooled samples within a given breed were above 0.97. Between 103 million and 110 million reads were mapped to the sheep reference genome for the STH and GAM sheep. Over 75% of the clean reads were mapped to the sheep reference genome, and, of these clean reads, more than 95% were only mapped to unique positions in the sheep reference genome. This provided a foundation for subsequent analyses.

**Figure 1:**
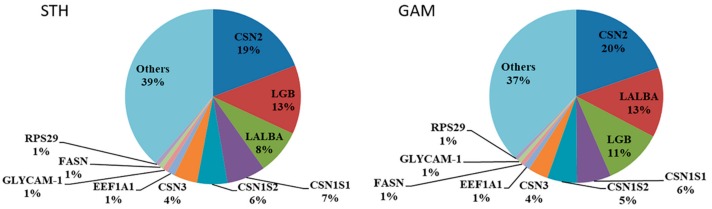
Pie charts of gene reads per kilobase per million (RPKM) in the Small-Tailed Han (STH) and Gansu Alpine Merino (GAM) sheep. A depiction of the genes that were the most highly expressed during lactation in the parenchyma using RNA-Seq analyses. The number below each gene is the proportion of total expression (the RPKM of the specific gene divided by the total RPKM of all genes in the sample).

The analysis revealed 13,520 genes with reads per kilobase per million (RPKM) > 0.01 in at least two samples from each library, and that were expressed in the parenchyma tissue at peak lactation in the STH and GAM sheep. Milk protein genes were highly expressed in both the STH and GAM sheep, with the expression of the beta-casein gene (*CSN2*) highest in both (Figure 1). In the STH sheep, the next most highly expressed gene was the beta-lactoglobulin gene (*LGB*), followed by the alpha-lactalbumin gene (*LALBA*), the α-S1 casein gene (*CSN1S1*), the α-S2 casein gene (*CSN1S2*), and the κ-casein gene (*CSN3*). In the GAM sheep *LALBA* was next most highly expressed, followed by *LGB*, *CSN1S1*, *CSN1S2*, and *CSN3*.

### 3.2. Identification of DEGs

**Figure 2:**
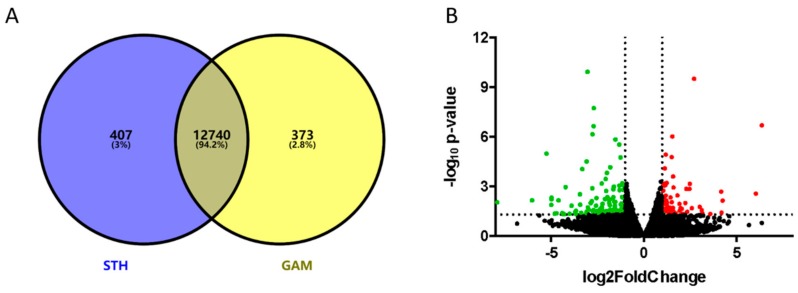
Differentially expressed gene distributions during lactation in the mammary gland of the Small-Tail Han (STH) and Gansu Alpine Merino (GAM) sheep. (**A**) Venn diagram summarizing the number of genes expressed only in the STH sheep, expressed only in the GAM sheep, and common to both groups. (**B**) Volcano plot comparing the change in gene expression of the STH and GAM sheep. The red and green dots indicate upregulated and downregulated genes in mammary gland of STH compared to the mammary gland of GAM, respectively (*p* < 0.05). The black dots represent genes that are not significantly different in the two breeds of sheep (*p* > 0.05).

In total, the expression of 12,740 genes was common to both breeds (Figure 2A). Of the 13,520 genes identified, 407 were only expressed in the STH sheep, while 373 genes were only expressed in the GAM sheep. Using the DESeq method, the comparison of gene expression profiles between the STH and GAM sheep allowed the identification of a total of 217 DEGs (Figure 2B).

Seventy-four DEGs (of which 67 were identified genes), were more abundant in the STH sheep than in the GAM sheep (Supplementary MaterialsTable S1). The five genes that had more abundant expression in the STH sheep compared to the GAM sheep were the ubiquitin-fold modifier-conjugating enzyme 1 gene (UFC1; UniProt W5P6Q6), syntrophin gamma 1 gene (*SNTG1*), the gastric inhibitory polypeptide receptor gene (*GIPR*), the synuclein gamma gene (*SNCG*), and the sialidase 2 gene (*NEU2*), with 82.7, 66.2, 19.1, 18.3, and 18.1-fold increases in expression respectively.

One hundred and forty three DEGs (of which 115 were identified genes), were less abundant in the STH sheep (Supplementary MaterialsTable S2). The five genes that had less abundant expression in the STH sheep compared to the GAM sheep were *RNF122*, a gene for an uncharacterized (but potentially transmembrane) protein (UniProt W5NQG5), another uncharacterized protein (which may have ATPase activity) gene (*W5NSR6*), the shisa family member 6 gene (*SHISA6*), the taxilin beta (*TXLNB*) gene, and the granzyme K gene (*GZMK*), with 250.0, 66.7, 38.5, 32.3 and 32.2-fold decreases in expression, respectively. 

### 3.3. GO Enrichment and KEGG Pathway Analyses

**Figure 3:**
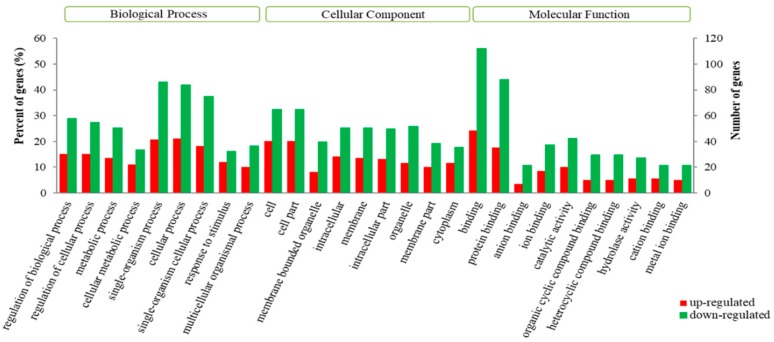
Gene ontology (GO) classification of the differentially expressed genes (DEGs). The GO annotation included biological process (BP), cellular component (CC) and molecular function (MF). Ten GO terms with the highest number of DEGs are illustrated for each category. The red indicates the GO terms that are enriched for upregulated genes in the mammary gland of the STH sheep, compared to the mammary gland of the GAM sheep. The green color indicates the GO terms that are enriched for downregulated genes in the mammary gland of the STH sheep, compared to the GAM sheep. The Y-axis annotation on the left indicates the percentage of DEGs involved in the GO-term, divided by the total number of DEGs, and the Y axis annotation on the right side represents the number of DEGs involved in the GO-term.

The results of the GO enrichment analysis gave a more comprehensive understanding of the function of the DEGs. These DEGs were enriched for 1117 biological process (GO-BP) terms, 133 cellular component (GO-CC) terms and 203 molecular functional (GO-MF) terms. While many other DEGs were enriched in functional GO terms, the differences were not significant (*p* > 0.05) due to there being a lower number of DEGs compared to the larger total number of genes in the specific GO terms. In the GO-BP, the most enriched GO terms were single-organism process (*p* = 0.181), cellular process (*p* = 0.192) and single-organism cellular process terms (*p* = 0.215). In the GO-CC, the most enriched GO terms were cell (*p* = 0.131), cell part (*p* = 0.213) and intracellular terms (*p* = 0.236). In the GO-MF, the most enriched GO terms were binding (*p* = 0.082), protein binding (*p* = 0.093), and catalytic activity terms (*p* = 0.115, Figure 3).

The KEGG analysis of the 217 DEGs revealed 84 enriched pathways, with the pathways of hematopoietic cell lineage (corrected *p* = 0.012), oxytocin signaling (corrected *p* = 0.018), neuroactive ligand–receptor interaction (corrected *p* = 0.031), phenylalanine, tyrosine and tryptophan biosynthesis (corrected *p* = 0.034), and cyclic adenosine monophosphate (cAMP) signaling (corrected *p* = 0.048) (Table 3).

### 3.4. Validation of Selected DEGs Using RT-qPCR

**Figure 4:**
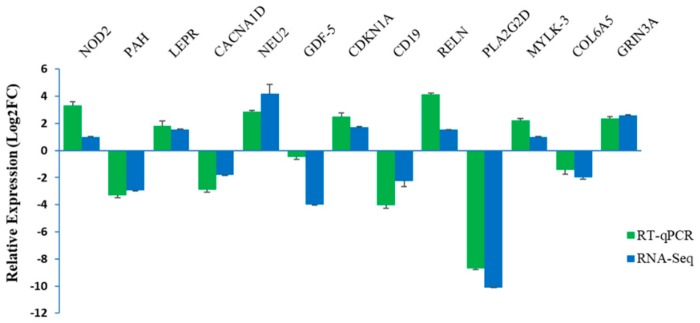
Comparison of gene expression levels measured using RNA-Seq and RT-qPCR. The relative expression levels of both the RT-qPCR and RNA-Seq were expressed as Log2fold change (FC), which is defined as the ratio of the expression level in the STH sheep to that in the GAM sheep for a specific gene. The expression values for the RT-qPCR are given relative to the expression levels of the *β-actin* and *GAPDH* gene. The error bars are a representation of the variation in the three separate sheep studied per group.

Thirteen DEGs were randomly selected for the validations, included seven upregulated genes, but six downregulated genes in the STH sheep compared to the GAM sheep. Their expression levels were calibrated relative to *β-actin* and *GAPDH* gene expression, whose stability in the experimental conditions employed, were found to be stable. These RT-qPCR (Figure 4) data confirmed the accuracy of the RNA-Seq data to quantify the gene expressions in the parenchyma of the ovine mammary gland.

## 4. Discussion

The aim of this study was to identify genes that might be associated with variation in milk yield by comparing two breeds of sheep. A total of 13,520 genes were found to be expressed in the parenchyma of the mammary gland. This compares with the results of Paten et al. [8], who described 10,096 genes that were expressed during lactation in the mammary glands of New Zealand Romney sheep, but we found more low-abundance expressed genes. Paten et al. [8] found genes with high levels of expression that were involved in milk fat synthesis, in ion transport, in protein synthesis, and in key signal transduction pathways, including the JAK-STAT and PI3K-Akt pathways. However, their study was a comparison of gene expression in mammary parenchymal tissue in late pregnancy versus expression in early lactation, while in our study we compared two breeds of sheep with different phenotypes for milk production traits.

Suárez-Vega et al. [9] studied milk somatic cells (MSC) and revealed an average of 16,757 and 16,897 unique expressed genes in Spanish Assaf and Churra sheep, respectively. This is comparable to the 13,520 genes reported here, despite the different source of RNA. Suárez-Vega et al. [9] suggested that MSCs are a representative source of RNA from mammary gland tissue, and based this suggestion on the observations of Canovas et al. [22] in cattle. In a more recent study using RNA-sequencing with MSCs, Suárez-Vega et al. [23] analyzed eight ewes, revealing 57,795 variants in the regions harboring quantitative trait loci (QTL) for milk yield, protein percentage and fat percentage, of which 21.44% were novel variants. In a subsequent functional enrichment analysis of the genes positioned within selected QTL, the KEGG pathway with the highest enrichment was ‘protein processing in endoplasmic reticulum’. Additionally, 504 and 1063 variants were identified in the genes encoding principal milk proteins and molecules involved in lipid metabolism, respectively, and of these variants, 20 sequence variations potentially affected protein function [23].

As might be expected, we found high levels of expression of the casein genes including *CSN2*, *CSN1S1*, *CSN1S2*, and *CSN3* (κ-casein), along with *LALBA* and *LGB*, with the β-casein gene (*CSN2*) having the highest level of expression in both breeds. Suárez-Vega et al. [10] also found casein genes and whey protein genes were highly expressed at all stages of ovine lactation (Days 10, 50, 120 and 150) in the Assaf and Churra sheep, and in both *CSN2* was also the most highly expressed casein gene. However, in contrast to our findings, Suárez-Vega et al. [10] revealed the κ-casein gene was the next most commonly expressed in both Spanish breeds, followed by *CSN1S1* and *CSN1S2*. The lactoglobulin genes *LGB* and *LALBA*, were expressed too, but at lower levels than the caseins. After the expressed caseins and lactoglobulins, Suárez-Vega et al. [10] revealed that the gene for the glycosylation-dependent cell adhesion molecule 1 (*GLYCAM-1*) was the next most commonly expressed in the MSCs, and in both the Churra and Assaf breeds. This gene was also expressed in both the Chinese breeds studied here.

In goats, Crisà et al. [24] studied MSCs and compared gene expression in colostrum with 120-days-old milk. They found *CSN2* to be the most highly expressed gene in both the colostrum and 120-days-old milk, but the next most highly expressed gene was *LGB* in colostrum and *CSN3* in 120-days-old milk. In cattle, Wickramasinghe et al. [6] also revealed *LGB* and *CSN2* to be highly expressed in early lactation, but with expression decreasing as lactation progressed.

Together these findings in other ruminants, along with those of Suárez-Vega et al. [10] in sheep, suggest that the balance between the expression of the casein and lactoglobulin genes changes as lactation progresses. This contrasts our findings, where in day 20 milk in both Chinese breeds, both *LGB* and *LALBA* are still expressed at higher levels. Whether this is a function of breed or environment, or both, cannot be ascertained.

Of the top five upregulated genes in the STH sheep compared to the GAM sheep, little could be concluded as to how these genes might affect milk traits. *UFC1* is one of the ubiquitin-like (UBL) post-translational modifiers that are covalently linked to most, if not all, target protein(s) through an enzymatic cascade analogous to ubiquitylation. Why it might be so greatly increased in expression cannot be ascertained. Syntrophin gamma 1 is a dystrophin-associated protein. The gene for syntrophin gamma 1 (*SNTG1*) was one of the top genes associated with more than five phenotypes for human pulmonary diseases [25], and it has been identified as a candidate gene for human idiopathic scoliosis [26]. Bovine *SNTG1* is found on chromosome 14 and has been associated with longevity in Fleckvieh cattle [27].

Of the top five downregulated genes in the STH sheep compared to the GAM sheep, the gene *RNF122* was the most downregulated. This is a gene for an uncharacterized, but potentially transmembrane protein that may be involved in basic processes such as protein–protein and protein–DNA interactions [28]. It may therefore play a role in cell viability, and it has been reported to have a role in immune response. The gene that was next most downregulated, was another uncharacterized protein (UniProt W5NSR6). Together, little can be said about how or why these genes may be affecting lactation.

Other DEGs of interest, which might be affecting lactation and under-pinning the differences between the STH and GAM sheep, include two upregulated DEGs (*DLL4* and *RELN*) and one downregulated DEG (*WNT5A*) in the mammary gland of the STH sheep. Previous studies have reported that *DLL4* produces a ligand in the notch-signaling pathway and plays an important role in the proliferation and regeneration of mammary gland stem cells [29]. The protein produced by *RELN* is involved in the regulation of mammary gland morphogenesis [30]. Suárez-Vega [9] also found that *RELN* was upregulated in 150-day MSC in Assaf sheep compared to that in 10-day MSC. In mice, the gene *WNT5A* is required for proper mammary gland development and is capable of inhibiting ductal extension and lateral branching in the mouse mammary gland [31].

The GO analyses results provided no support for us to better understand the differences between the STH and GAM sheep. However, Suárez-Vega et al. [9] revealed that single-organism processes were enriched in MSC of Churra sheep (a lower milk-yield breed) compared to Assaf sheep (a higher milk-yield breed). In a study of goat colostrum MSCs, the most common GO-MF terms were associated with protein binding [24] and Ji et al. [32] revealed that the main GO terms included cellular process, cells, cell part, binding and catalytic activity in lactating dairy goat mammary gland tissues. It is therefore suggested that these biological processes, cellular components and molecular functions are important for lactation and mammary gland development in domestic animals. While the milk yield of the mammary gland is correlated with the ability of the mammary gland alveolus to secrete milk, cellular processes, such as growth, maintenance, apoptosis and signaling, will underpin this activity.

When comparing the two sheep breeds in this study, the most prominent difference (*p* < 0.05) in the DEGs was for hematopoietic cell lineages. The cluster of differentiation (CD) family members *CD22*, *CD19* and *CD13* were downregulated in the mammary gland of the STH sheep compared to the mammary gland of the GAM sheep. These genes inhibit B-cell signaling by negatively regulating B-cell receptor (BCR) activation [33]. It has been reported that the expression level of others CDs (*CD74* and *CD24*) was different at different stages of lactation in bovine MSC (lactation at 15 days, peak-lactation and late-lactation) [6]. How these activities affect milk production, given the central role of the CDs in immunity, cannot be ascertained without further study, but it is tempting to suggest that they might be associated with the important role milk has in the acquisition of passive immunity by neonates.

Two of the upregulated genes (*MYLK-3* and *CDKN1A*) and two of the downregulated genes (*RGS2* and *CACNA1D*) are part of the oxytocin-signaling pathway. Its best-established roles are in the stimulation of uterine contractions during parturition and subsequently in milk release during lactation. In a study of yaks, some upregulated DEGs during lactation were from the oxytocin-signaling pathway, when compared to non-lactating yaks [7].

Taken together these results may assist in the selection of candidate genes that have functional roles in the ovine mammary gland, while also possibly explaining the difference in performance between the STH and GAM sheep.

## 5. Conclusions

This study describes the transcriptome of mammary gland parenchyma of GAM and STH sheep. The differences in the gene expression profiles, although small, might serve to identify genes that have functional role in lactation and explain the differences in production characteristics that exist between these two breeds.

## Figures and Tables

**Table 1:** Primers used for the RT-qPCR analyses.

Gene	Forward (5′→3′)	Reverse (5′→3′)
*NOD2*	GAATTACCGGTCCCATTGGC	ACACTTCTTCCAGGCACAGA
*PLA2G2D*	AACCCAGAGATGCCACAGAC	AAGCCAACGTCTTGTCACAG
*LEPR*	TGTTGCTTTGGAGTGAGGA	TCCAGTGTGCACCTGTTTGT
*NEU2*	GACGAGCAAGAAGGATGAGC	CGGGGATGGCAATGAAGAAG
*CDKN1A*	GAGAGCGATGGAACTTCGAC	AGTGGTCCTCCTGAGACGTG
*COL6A5*	GAGACCATCGCAGGGGATAA	ACCATGTCAGAGCCATCACA
*RELN*	ACTCTGGGCCAAACTGCTAT	TTGTCTCACTGTGGATCCCC
*MYLK-3*	GCTGGCCAGAAGATACAAGC	CGGGAACGAGACAAACTCAT
*GR1N3A*	GCAAATATGGAGCCTGGAAA	CTGGCTTCGTGCAGTATTGA
*PAH*	CGCTGTCCAGGAGTATACGA	TTGTGGCAGCAAAGTTCCTC
*CACNA1D*	TTCCCAGCTCAACAAATGCC	TGCCCGTTTTCAGACACAAG
*GDF-5*	GGGCTGGGATGACTGGATTA	GGCTGAGTCGATGAAGAGGA
*CD19*	AGATGCAGCTGAAGGTCACT	CAGGGAAGTCAGGCAGAAGA
*β-actin*	AGCCTTCCTTCCTGGGCATGGA	GGACAGCACCGTGTTGGCGTAGA
*GAPDH*	ATCTCGCTCCTGGAAGATG	TCGGAGTGAACGGATTCG

**Table 2:** Summary of the RNA-Seq analyses after mapping to the reference genome.

Sample	Useful Reads	Map Event	Map Reads	Map Reads (%)	Multiple Reads	Multiple Reads (%)	Unique Reads	Unique Reads (%)
GAM-1	141244574	119373530	110324120	78.11	3934715	3.57	106389405	96.43
GAM-2	139563102	118260385	107968116	77.36	4219681	3.91	103748435	96.09
GAM-3	139407058	117075040	106817466	77.62	4707008	4.41	102110458	95.59
STH-1	140828556	117044888	108043045	76.72	3664975	3.39	104378070	96.61
STH-2	136465766	113483944	103333734	75.72	4688735	4.54	98644999	95.46
STH-3	136990336	111826264	103170887	75.31	3926392	3.81	99244495	96.19

**Table 3:** Kyoto Encyclopedia of Genes and Genomes (KEGG) pathway analysis for DEGs identified in comparing the STH vs. GAM sheep.

KEGG Pathway ^1^	Upregulated Genes	Down-Regulated Genes	Corrected *p*-Value
Hematopoietic cell lineage		*CD22, CD19, CD13*	0.012
Oxytocin signaling pathway	*MYLK-3, CDKN1A*	*RGS2, CACNA1D*	0.018
Neuroactive ligand-receptor interaction	*GIPR, LEPR, GRIN3A*	*HTR4, LPAR4*	0.031
Phenylalanine, tyrosine and tryptophan biosynthesis		*PAH*	0.034
cAMP signaling pathway	*GIPR, GRIN3A*	*CACNA1D, HTR4*	0.048

^1^ Five different KEGG pathways were identified (corrected *p*-value < 0.05).

## Supplementary Materials

The following are available online at https://www.mdpi.com/2073-4425/10/10/781/s1, Table S1: The upregulated genes in the mammary gland of the STH sheep compared with the GAM sheep, Table S2: The downregulated genes in the mammary gland of the STH sheep compared with the GAM sheep.
