# Supplementary material for: Transcriptome Profile Analysis of Mammary Gland Tissue from Two Breeds of Lactating Sheep
Source: Genes (Basel). 2019 Oct 8;10(10):781. doi: 10.3390/genes10100781 (PMC6826511; doi:10.3390/genes10100781)
Supplement: Supplementary file 1 [file genes-10-00781-s001.pdf]

Table S1. The up-regulated genes in the mammary gland of the STH sheep compared with the GAM sheep.

Gene ID	Gene name	Base mean	Base mean-GAM	Base mean-STH	Fold change	log_2(Fold change)	P value	Description
ENSOARG00000005699	UFC1	21.80852558	0.521053764	43.0959974	82.70931	6.369977831	2.06E-07	Uncharacterized protein [Source:UniProtKB/TrEMBL;Acc:W5P6Q6]
ENSOARG00000013119	SNTG1	8.758370481	0.260526882	17.25621408	66.23583	6.049539927	0.0027479	Syntrophin, gamma 1 [Source:HGNCSymbol;Acc:HGNC:13740]
ENSOARG00000010160	GIPR	8.105104327	0.807424204	15.40278445	19.07645	4.253720534	0.0071514	Gastric inhibitory polypeptide receptor [Source:HGNC Symbol;Acc:HGNC:4271]
ENSOARG00000000714	SNCG	5.714185855	0.591922793	10.83644892	18.3072	4.194339244	0.0387692	Synuclein, gamma (breast cancer-specific protein 1) [Source:HGNC Symbol;Acc:HGNC:11141]
ENSOARG00000019344	NEU2	10.85689932	1.138820115	20.57497852	18.06693	4.175279139	0.0020472	Sialidase 2 (cytosolic sialidase) [Source:HGNCSymbol;Acc:HGNC:7759]
ENSOARG00000000724	-	11.29625806	1.73219007	20.86032605	12.04275	3.59009256	0.0464916	-
ENSOARG00000006532	KCNK4	7.882984721	1.587557688	14.17841175	8.930958	3.158815011	0.0411659	Potassium channel, subfamily K, member 4 [Source:HGNC Symbol;Acc:HGNC:6279]
ENSOARG00000012671	RNASE1	31.42267299	6.4483905	56.39695548	8.745896	3.128606265	0.0271619	Ribonuclease pancreatic [Source:UniProtKB/Swiss-Prot;Acc:P67927]
ENSOARG00000020334	SPX	10.89263026	2.394981892	19.39027863	8.096211	3.017246881	0.0172611	Spexin hormone [Source:HGNC Symbol;Acc:HGNC:28139]
ENSOARG00000005894	CDH20	163.7153348	43.17893704	284.2517326	6.583111	2.718769509	3.22E-10	Cadherin 20, type 2 [Source:HGNCSymbol;Acc:HGNC:1760]
ENSOARG00000007440	GRIN3A	12.80810009	3.646802182	21.96939799	6.024291	2.590791489	0.0207721	Glutamate receptor, ionotropic, N-methyl-D-aspartate 3A [Source:HGNC Symbol;Acc:HGNC:16767]
ENSOARG00000002539	MYRIP	32.10231044	9.740847538	54.46377334	5.591277	2.483177728	0.0007048	Myosin VIIA and Rab interacting protein [Source:HGNC Symbol;Acc:HGNC:19156]
ENSOARG00000015190	-	243.9883915	74.82303815	413.1537449	5.521745	2.465124293	0.0014163	Uncharacterized protein [Source:UniProtKB/TrEMBL;Acc:W5Q0R2]
ENSOARG00000019726	RNASE12	26.1924995	8.634532625	43.75046637	5.066918	2.341108402	0.0340688	Ribonuclease, RNase A family, 12 (non-active) [Source:HGNC Symbol;Acc:HGNC:24211]
ENSOARG00000011248	AMPD3	132.7364406	44.87262333	220.6002579	4.916144	2.297527043	0.0013823	Adenosine monophosphate deaminase 3 [Source:HGNCSymbol;Acc:HGNC:470]
ENSOARG00000017812	CASKIN1	20.59876909	7.161996442	34.03554173	4.752242	2.248608366	0.0101967	CASK interacting protein 1 [Source:HGNCSymbol;Acc:HGNC:20879]
ENSOARG00000006766	ASB5	17.33531992	6.563412005	28.10722784	4.282411	2.098423267	0.0448267	Ankyrin repeat and SOCS box containing 5 [Source:HGNC Symbol;Acc:HGNC:17180]
ENSOARG00000003279	KCNIP1	18.64209439	7.252920379	30.03126839	4.140576	2.049831493	0.0225371	Kv channel interacting protein 1 [Source:HGNCSymbol;Acc:HGNC:15521]
ENSOARG00000013948	GABRA1	20.44878247	8.225606041	32.67195891	3.971982	1.989859075	0.0206184	Gamma-aminobutyric acid (GABA) A receptor, alpha 1 [Source:HGNC Symbol;Acc:HGNC:4075]
ENSOARG00000013223	RHBDL1	26.44419551	10.93953355	41.94885747	3.834611	1.939080293	0.0293236	Rhomboid, veinlet-like 1 (Drosophila) [Source:HGNCSymbol;Acc:HGNC:10007]
ENSOARG00000002469	-	307.3492226	127.9895681	486.708877	3.802723	1.927032861	0.0113325	-
ENSOARG00000009458	-	34.3393511	15.1839772	53.494725	3.523104	1.816846907	0.003524	Uncharacterized protein [Source:UniProtKB/TrEMBL;Acc:W5PI81]
ENSOARG00000013636	CDKN1A	117.1247866	54.59408359	179.6554896	3.29075	1.718416501	0.0426504	*Ovis aries* cyclin-dependent kinase inhibitor 1A (p21, Cip1) (CDKN1A), mRNA

								[Source:RefSeqmRNA;Acc:NM_001161880]
ENSOARG00000018585	NOX5	114.1739114	56.44876439	171.8990584	3.045223	1.606547735	0.0011552	NADPH oxidase, EF-hand calcium binding domain 5 [Source:HGNC Symbol;Acc:HGNC:14874]
ENSOARG00000009381	PLA2G2C	207.5902297	103.5560238	311.6244356	3.009235	1.589396888	0.0002552	Phospholipase A2, group IIC [Source:HGNCSymbol;Acc:HGNC:9032]
ENSOARG00000017483	HOXD4	27.44644248	13.75357216	41.13931279	2.991173	1.580711322	0.0276371	Homeobox D4 [Source:HGNC Symbol;Acc:HGNC:5138]
ENSOARG00000007618	IL3RA	59.76831789	30.00970654	89.52692924	2.983266	1.576892492	0.0307495	Interleukin 3 receptor, alpha (low affinity) [Source:HGNC Symbol;Acc:HGNC:6012]
ENSOARG00000016907	UNC5C	210.7270196	105.9135149	315.5405244	2.979228	1.574938607	0.0195074	Unc-5 homolog C (C. elegans) [Source:HGNCSymbol;Acc:HGNC:12569]
ENSOARG00000007828	SNTA1	97.91585228	49.41387095	146.4178336	2.963092	1.567103302	0.041657	Syntrophin, alpha 1 [Source:HGNCSymbol;Acc:HGNC:11167]
ENSOARG00000009197	ACADSB	501.5286338	256.2069868	746.8502808	2.915027	1.54350924	9.71E-07	Acyl-CoA dehydrogenase, short/branched chain [Source:HGNC Symbol;Acc:HGNC:91]
ENSOARG00000010394	LEPR	199.5704477	102.7141385	296.4267568	2.885939	1.529040897	0.0437272	*Ovis aries* leptin receptor (LEPR), mRNA [Source:RefSeq mRNA;Acc:NM_001009763]
ENSOARG00000007561	SNAP91	35.65156655	18.44530622	52.85782687	2.865652	1.518863373	0.0395895	Synaptosomal-associated protein, 91kDa [Source:HGNCSymbol;Acc:HGNC:14986]
ENSOARG00000012284	NIPAL4	43.77214505	22.71081772	64.83347238	2.85474	1.513359191	0.0089381	NIPA-like domain containing 4 [Source:HGNCSymbol;Acc:HGNC:28018]
ENSOARG00000000465	RELN	1302.762493	676.4612894	1929.063698	2.851699	1.511821499	0.0272581	Reelin [Source:HGNC Symbol;Acc:HGNC:9957]
ENSOARG00000014408	ADGRG2	234.3768852	122.0110494	346.7427209	2.841896	1.506853791	1.72E-05	G protein-coupled receptor 64 [Source:HGNCSymbol;Acc:HGNC:4516]
ENSOARG00000003813	C16orf71	31.80995958	16.56565942	47.05425974	2.84047	1.5061297	0.0353878	Chromosome 16 open reading frame 71 [Source:HGNCSymbol;Acc:HGNC:25081]
ENSOARG00000014150	-	34.95545746	18.24471521	51.6661997	2.831845	1.501742139	0.029363	Ribosomal protein [Source:UniProtKB/TrEMBL;Acc:W5PXK5]
ENSOARG00000011385	TMEM42	32.3427742	17.27983953	47.40570886	2.743411	1.455970987	0.0441557	Transmembrane protein 42 [Source:HGNCSymbol;Acc:HGNC:28444]
ENSOARG00000005911	INSC	85.5921101	46.01962223	125.164598	2.719809	1.443505517	0.0045958	Inscuteable homolog (Drosophila) [Source:HGNCSymbol;Acc:HGNC:33116]
ENSOARG00000014253	SLC7A11	188.0951449	102.9295745	273.2607153	2.654832	1.408620501	0.0192654	Anionic amino acid transporter light chain xCT;Uncharacterized protein [Source:UniProtKB/TrEMBL;Acc:H2EUS8]
ENSOARG00000020860	ALDH1A2	44.93049346	24.81970452	65.0412824	2.62055	1.389869763	0.0322404	Aldehyde dehydrogenase 1 family, member A2 [Source:HGNC Symbol;Acc:HGNC:15472]
ENSOARG00000003423	CYP1A1	28.09554305	15.80608504	40.38500106	2.555029	1.353339503	0.0378788	*Ovis aries* cytochrome P4501A1 (CYP1A1), mRNA [Source:RefSeq mRNA;Acc:NM_001129905]
ENSOARG00000004231	LRRC3B	40.04687079	22.53368014	57.56006144	2.554401	1.352985182	0.0298575	Leucine rich repeat containing 3B [Source:HGNCSymbol;Acc:HGNC:28105]
ENSOARG00000004563	NKIRAS1	56.19052069	32.95845277	79.42258861	2.409779	1.268900866	0.0160861	NFKB inhibitor interacting Ras-like 1 [Source:HGNCSymbol;Acc:HGNC:17899]
ENSOARG00000020932	C2orf40	69.93876543	41.27700407	98.60052678	2.388752	1.256257092	0.027124	Chromosome 2 open reading frame 40 [Source:HGNCSymbol;Acc:HGNC:24642]
ENSOARG00000009400	PLIN4	81.6804045	48.25896861	115.1018404	2.385087	1.254041914	0.0097078	Perilipin 4 [Source:HGNC Symbol;Acc:HGNC:29393]
ENSOARG00000005390	TMEM184A	359.6646113	213.9511258	505.3780968	2.362119	1.240081869	0.0006277	Transmembrane protein 184A [Source:HGNCSymbol;Acc:HGNC:28797]
ENSOARG00000002849	-	50.45038997	30.22396616	70.67681378	2.338436	1.225544011	0.0313438	-

ENSOARG00000003957	-	90.73659124	54.60370954	126.8694729	2.323459	1.216274103	0.007007	60S ribosomal protein L13 [Source:UniProtKB/TrEMBL;Acc:W5P1C7]
ENSOARG00000004943	FAM135B	958.7224716	584.4433729	1333.00157	2.280805	1.189543327	0.0036027	Family with sequence similarity 135, member B [Source:HGNC Symbol;Acc:HGNC:28029]
ENSOARG00000006407	EPHA5	1274.135407	777.3305922	1770.940222	2.278233	1.187915315	1.22E-05	EPH receptor A5 [Source:HGNC Symbol;Acc:HGNC:3389]
ENSOARG00000010183	CTTNBP2	812.066898	497.6082063	1126.52559	2.263881	1.178797903	0.0008262	*Ovis aries* cortactin binding protein 2 (CTTNBP2), mRNA [Source:RefSeq mRNA;Acc:NM_001195311]
ENSOARG00000013348	DAGLA	250.2245913	155.0233226	345.4258601	2.228219	1.155890814	0.0006696	Diacylglycerol lipase, alpha [Source:HGNCSymbol;Acc:HGNC:1165]
ENSOARG00000009705	WISP3	370.144582	230.1648537	510.1243103	2.216343	1.148181305	0.0065636	WNT1 inducible signaling pathway protein 3 [Source:HGNC Symbol;Acc:HGNC:12771]
ENSOARG00000005014	ABLIM3	619.5466386	385.6858839	853.4073932	2.212701	1.145808265	8.22E-05	Actin binding LIM protein family, member 3 [Source:HGNC Symbol;Acc:HGNC:29132]
ENSOARG00000020309	DLL4	422.9912531	268.2788893	577.7036169	2.15337	1.106595993	0.0009079	Delta-like 4 (Drosophila) [Source:HGNCSymbol;Acc:HGNC:2910]
ENSOARG00000006391	SERPINB5	131.2364501	83.44656764	179.0263326	2.145401	1.101247191	0.0048986	Serpin peptidase inhibitor, clade B (ovalbumin), member 5 [Source:HGNC Symbol;Acc:HGNC:8949]
ENSOARG00000000474	GJA4	100.5783395	64.17784845	136.9788306	2.134363	1.093805619	0.0110677	Gap junction protein, alpha 4, 37kDa [Source:HGNCSymbol;Acc:HGNC:4278]
ENSOARG00000018720	SNPH	162.7524299	104.2866969	221.2181628	2.12125	1.084914706	0.0051949	Syntaphilin [Source:HGNC Symbol;Acc:HGNC:15931]"
ENSOARG00000005655	MMP28	186.5021481	119.7454672	253.2588291	2.114976	1.080641519	0.0051728	Matrix metallopeptidase 28 [Source:HGNCSymbol;Acc:HGNC:14366]
ENSOARG00000003976	SH2D4A	279.9543332	180.2660656	379.6426009	2.106013	1.07451405	0.0065911	SH2 domain containing 4A [Source:HGNCSymbol;Acc:HGNC:26102]
ENSOARG00000006059	BEND7	198.1845551	127.637656	268.7314542	2.105425	1.074111174	0.0072055	BEN domain containing 7 [Source:HGNCSymbol;Acc:HGNC:23514]
ENSOARG00000001496	ZSWIM5	81.70204826	53.0454639	110.3586326	2.080454	1.056898195	0.0217781	Zinc finger, SWIM-type containing 5 [Source:HGNCSymbol;Acc:HGNC:29299]
ENSOARG00000008661	CCDC37	70.53922177	45.8135414	95.26490213	2.079405	1.056170701	0.0394244	Coiled-coil domain containing 37 [Source:HGNCSymbol;Acc:HGNC:26842]
ENSOARG00000008806	WIPF3	216.7401769	140.9682642	292.5120896	2.075021	1.053125844	0.0017936	WAS/WASL interacting protein family, member 3 [Source:HGNC Symbol;Acc:HGNC:22004]
ENSOARG00000008361	ARSD	123.3897061	80.46696874	166.3124435	2.066841	1.047427524	0.0471848	Arylsulfatase D [Source:HGNC Symbol;Acc:HGNC:717]
ENSOARG00000006153	PDGFA	85.00395759	55.5584316	114.4494836	2.059984	1.042633175	0.0342606	Platelet-derived growth factor alpha polypeptide [Source:HGNC Symbol;Acc:HGNC:8799]
ENSOARG00000009523	SLC25A15	561.6781781	367.3128888	756.0434674	2.058309	1.041459662	0.0006772	*Ovis aries* solute carrier family 25 (mitochondrialcarrier; ornithine transporter) member 15 (SLC25A15), nuclear gene encoding mitochondrial protein, mRNA [Source:RefSeq mRNA;Acc:NM_001127276]
ENSOARG00000001753	NQO2	155.2188589	101.5840059	208.8537119	2.05597	1.039819512	0.0122971	NAD(P)H dehydrogenase, quinone 2 [Source:HGNCSymbol;Acc:HGNC:7856]
ENSOARG00000004847	PDE10A	270.8689677	178.1933998	363.5445356	2.040168	1.02868821	0.0059556	Phosphodiesterase 10A [Source:HGNCSymbol;Acc:HGNC:8772]
ENSOARG00000001390	SRD5A3	111.5636771	73.64114391	149.4862104	2.029928	1.021428464	0.0110381	Steroid 5 alpha-reductase 3 [Source:HGNCSymbol;Acc:HGNC:25812]
ENSOARG00000015706	MYLK3	89.00664293	59.15717403	118.8561118	2.009158	1.006591051	0.0214293	Myosin light chain kinase 3 [Source:HGNCSymbol;Acc:HGNC:29826]

ENSOARG00000017441	NOD2	1147.689563	763.837986	1531.541141	2.00506	1.003645549	0.013456	Nucleotide-binding oligomerization domaincontaining 2 [Source:HGNC Symbol;Acc:HGNC:5331]
ENSOARG00000011450	FEZ1	72.1825533	48.09660147	96.26850512	2.001566	1.001128932	0.0436743	Fasciculation and elongation protein zeta 1 (zyginI) [Source:HGNC Symbol;Acc:HGNC:3659]

Table S2. The down-regulated genes in the mammary gland of the STH sheep compared with the GAM sheep.

Gene ID	Gene name	Base mean	Base mean-GAM	Base mean-STH	Fold change	log_2(Fold change)	P value	Description
ENSOARG00000000415	RNF122	38.04557	75.77284	0.318297	0.004201	-7.89516	0.009321	Uncharacterized protein [Source:UniProtKB/TrEMBL;Acc:W5NQG5]
ENSOARG00000001169	-	121.4945	239.2925	3.696418	0.015447	-6.0165	0.007016	Uncharacterized protein [Source:UniProtKB/TrEMBL;Acc:W5NSR6]
ENSOARG00000014784	SHISA6	103.8983	202.4546	5.342038	0.026386	-5.24406	1.05E-05	Shisa family member 6 [Source:HGNCSymbol;Acc:HGNC:34491]
ENSOARG00000001219	TXLNB	6.231411	12.08125	0.381574	0.031584	-4.98466	0.013157	Taxilin beta [Source:HGNC Symbol;Acc:HGNC:21617]
ENSOARG00000007987	GZMK	15.68494	30.40414	0.965738	0.031763	-4.97649	0.005976	Granzyme K (granzyme 3; tryptase II) [Source:HGNCSymbol;Acc:HGNC:4711]
ENSOARG00000004467	SCN7A	66.86946	129.5794	4.159552	0.0321	-4.96126	0.004872	Sodium channel, voltage-gated, type VII, alphasubunit [Source:HGNC Symbol;Acc:HGNC:10594]
ENSOARG00000020250	C12orf60	4.63943	8.956947	0.321913	0.03594	-4.79827	0.044571	Chromosome 12 open reading frame 60 [Source:HGNCSymbol;Acc:HGNC:28726]
ENSOARG00000001488	HIST3H3	5.134687	9.8878	0.381574	0.03859	-4.69561	0.041961	Histone cluster 3, H3 [Source:HGNCSymbol;Acc:HGNC:4778]
ENSOARG00000013873	RGS20	7.992227	15.34786	0.636593	0.041478	-4.59152	0.006997	Regulator of G-protein signaling 20 [Source:HGNCSymbol;Acc:HGNC:14600]
ENSOARG00000014427	-	10.4941	20.0333	0.95489	0.047665	-4.39092	0.041148	Uncharacterized protein [Source:UniProtKB/TrEMBL;Acc:W5PYE6]
ENSOARG00000018798	DNAJC22	11.20955	21.33766	1.081445	0.050682	-4.30237	0.048476	DnaJ (Hsp40) homolog, subfamily C, member 22 [Source:HGNC Symbol;Acc:HGNC:25802]
ENSOARG00000018245	GCNT7	18.33724	34.88593	1.788547	0.051268	-4.28579	0.047535	Glucosaminyl (N-acetyl) transferase family member 7 [Source:HGNC Symbol;Acc:HGNC:16099]
ENSOARG00000008552	ARHGEF33	625.8271	1187.287	64.36672	0.054213	-4.20521	0.001114	Rho guanine nucleotide exchange factor (GEF) 33 [Source:HGNC Symbol;Acc:HGNC:37252]
ENSOARG00000012471	GDF5	5.448809	10.25379	0.643825	0.062789	-3.99335	0.044722	Growth differentiation factor 5 [Source:HGNCSymbol;Acc:HGNC:4220]
ENSOARG00000020452	NYAP2	8.040989	15.06381	1.018167	0.06759	-3.88704	0.014675	Neuronal tyrosine-phosphorylated phosphoinositide-3-kinase adaptor 2 [Source:HGNC Symbol;Acc:HGNC:29291]
ENSOARG00000015341	TSPAN19	6.736823	12.50791	0.965738	0.07721	-3.69507	0.036955	Tetraspanin 19 [Source:HGNC Symbol;Acc:HGNC:31886]
ENSOARG00000001543	-	6.58028	12.19482	0.965738	0.079192	-3.65849	0.046455	Uncharacterized protein [Source:UniProtKB/TrEMBL;Acc:W5NTW6]
ENSOARG00000017103	MATN3	11.30406	20.93889	1.669224	0.079719	-3.64894	0.030064	Matrilin 3 [Source:HGNC Symbol;Acc:HGNC:6909]
ENSOARG00000005049	CP	13.54586	24.80227	2.289445	0.092308	-3.4374	0.002972	*Ovis aries* ceruloplasmin (ferroxidase) (CP), mRNA [Source:RefSeq mRNA;Acc:NM_001009733]
ENSOARG00000011773	-	21.02865	38.4802	3.577095	0.092959	-3.42726	0.012686	Uncharacterized protein [Source:UniProtKB/TrEMBL;Acc:W5PQ95]
ENSOARG00000001542	GIMAP2	9.438273	17.21455	1.661993	0.096546	-3.37264	0.039586	GTPase, IMAP family member 2 [Source:HGNCSymbol;Acc:HGNC:21789]
ENSOARG00000005582	C5	4570.469	8303.358	837.5795	0.100872	-3.3094	9.05E-05	Complement component 5 [Source:HGNCSymbol;Acc:HGNC:1331]
ENSOARG00000004653	CNDP1	7.131544	12.80007	1.463019	0.114298	-3.12913	0.049564	Carnosine dipeptidase 1 (metallopeptidase M20family) [Source:HGNC Symbol;Acc:HGNC:20675]
ENSOARG00000002875	-	177.0609	316.7276	37.39426	0.118064	-3.08235	3.14E-05	-

ENSOARG00000013047	RAB19	9.906422	17.71323	2.099612	0.118534	-3.07663	0.039311	RAB19, member RAS oncogene family [Source:HGNCSymbol;Acc:HGNC:19982]
ENSOARG00000006960	FCRL1	14.16128	25.31326	3.009304	0.118883	-3.07239	0.033629	Fc receptor-like 1 [Source:HGNCSymbol;Acc:HGNC:18509]
ENSOARG00000004675	SCN9A	113.6526	202.4891	24.81609	0.122555	-3.0285	1.23E-10	Sodium channel, voltage-gated, type IX, alphasubunit [Source:HGNC Symbol;Acc:HGNC:10597]
ENSOARG00000001271	ENTPD8	27.42813	48.78885	6.067423	0.124361	-3.0074	0.018941	Ectonucleoside triphosphate diphosphohydrolase 8 [Source:HGNC Symbol;Acc:HGNC:24860]
ENSOARG00000015870	PAH	10.05082	17.8122	2.289445	0.128532	-2.9598	0.026041	Phenylalanine hydroxylase [Source:HGNCSymbol;Acc:HGNC:8582]
ENSOARG00000002032	-	27.81327	48.86638	6.760163	0.13834	-2.85371	0.010063	-
ENSOARG00000003072	-	16.54116	28.90831	4.174015	0.144388	-2.79198	0.00786	Uncharacterized protein [Source:UniProtKB/TrEMBL;Acc:W5NYM2]
ENSOARG00000004062	RAB38	22.67112	39.53707	5.805172	0.146829	-2.7678	0.00131	RAB38, member RAS oncogene family [Source:HGNCSymbol;Acc:HGNC:9776]
ENSOARG00000001114	-	259.4486	452.1217	66.77547	0.147694	-2.75932	7.14E-07	Uncharacterized protein [Source:UniProtKB/TrEMBL;Acc:W5NSL4]
ENSOARG00000004007	LIPG	1115.759	1940.39	291.1283	0.150036	-2.73662	0.032912	Lipase, endothelial [Source:HGNCSymbol;Acc:HGNC:6623]
ENSOARG00000004789	HTR4	11.98899	20.79331	3.184673	0.153159	-2.7069	0.026877	5-hydroxytryptamine (serotonin) receptor 4, Gprotein-coupled [Source:HGNC Symbol;Acc:HGNC:5299]
ENSOARG00000001282	-	244.9585	424.3495	65.56747	0.154513	-2.6942	2.32E-07	Uncharacterized protein [Source:UniProtKB/TrEMBL;Acc:W5NT39]
ENSOARG00000002425	-	122.1622	211.5089	32.81537	0.155149	-2.68827	1.88E-08	-
ENSOARG00000014467	EOMES	30.78865	52.67063	8.90668	0.169101	-2.56404	0.014572	Eomesodermin [Source:HGNC Symbol;Acc:HGNC:3372]
ENSOARG00000015375	ERC2	18.72002	31.96231	5.477734	0.171381	-2.54472	0.00502	ELKS/RAB6-interacting/CAST family member 2 [Source:HGNC Symbol;Acc:HGNC:31922]
ENSOARG00000014165	RIPPLY3	11.22614	19.14276	3.309522	0.172886	-2.5321	0.031304	Ripply transcriptional repressor 3 [Source:HGNCSymbol;Acc:HGNC:3047]
ENSOARG00000016321	KAZALD1	12.46325	21.03473	3.891776	0.185017	-2.43427	0.029431	Kazal-type serine peptidase inhibitor domain 1 [Source:HGNC Symbol;Acc:HGNC:25460]
ENSOARG00000019354	SLC7A8	20.65012	34.74277	6.557472	0.188744	-2.4055	0.045049	Solute carrier family 7 (amino acid transporterlight chain, L system), member 8 [Source:HGNCSymbol;Acc:HGNC:11066]
ENSOARG00000015313	SPOCK1	17.7569	29.705	5.808787	0.195549	-2.3544	0.02379	Sparc/osteonectin, cwcv and kazal-like domainsproteoglycan (testican) 1 [Source:HGNCSymbol;Acc:HGNC:11251]
ENSOARG00000008310	TTC29	11.79714	19.57956	4.014715	0.205046	-2.28598	0.045261	Tetratricopeptide repeat domain 29 [Source:HGNCSymbol;Acc:HGNC:29936]
ENSOARG00000017006	-	123.4262	204.4746	42.37784	0.207252	-2.27054	0.034817	Uncharacterized protein [Source:UniProtKB/TrEMBL;Acc:W5Q6B6]
ENSOARG00000001710	CD19	36.22469	59.93657	12.5128	0.208767	-2.26003	0.000669	CD19 molecule [Source:HGNC Symbol;Acc:HGNC:1633]
ENSOARG00000011787	-	23.00508	38.01487	7.995282	0.21032	-2.24934	0.018451	Uncharacterized protein [Source:UniProtKB/TrEMBL;Acc:W5PQA4]
ENSOARG00000006104	MB21D1	19.51994	32.14242	6.897464	0.214591	-2.22034	0.011377	Mab-21 domain containing 1 [Source:HGNCSymbol;Acc:HGNC:21367]
ENSOARG00000017651	-	15.77523	25.79218	5.758267	0.223256	-2.16323	0.032077	-
ENSOARG00000014329	ACOXL	15.01037	24.39284	5.627893	0.230719	-2.11579	0.041309	Acyl-CoA oxidase-like [Source:HGNCSymbol;Acc:HGNC:25621]
ENSOARG00000017181	CCDC158	27.82525	45.04556	10.60493	0.235427	-2.08665	0.005854	Coiled-coil domain containing 158 [Source:HGNCSymbol;Acc:HGNC:26374]

ENSOARG00000016789	NIPAL1	56.74119	91.44799	22.03438	0.24095	-2.0532	0.000358	NIPA-like domain containing 1 [Source:HGNCSymbol;Acc:HGNC:27194]
ENSOARG00000013767	IGSF9B	26.10002	42.00219	10.19784	0.242793	-2.0422	0.041731	Immunoglobulin superfamily, member 9B [Source:HGNCSymbol;Acc:HGNC:32326]
ENSOARG00000021015	CLEC7A	62.57862	100.6776	24.47961	0.243149	-2.04009	0.033461	Uncharacterized protein [Source:UniProtKB/TrEMBL;Acc:W5QIN1]
ENSOARG00000015616	ABCA13	23.92234	38.46072	9.383973	0.243989	-2.03511	0.045037	ATP-binding cassette, sub-family A (ABC1), member13 [Source:HGNC Symbol;Acc:HGNC:14638]
ENSOARG00000005785	MYO16	15.11541	24.2755	5.955331	0.245323	-2.02725	0.043467	Myosin XVI [Source:HGNC Symbol;Acc:HGNC:29822]
ENSOARG00000008972	AGR2	180.7417	290.0853	71.39808	0.246128	-2.02252	0.001904	Anterior gradient 2 [Source:HGNCSymbol;Acc:HGNC:328]
ENSOARG00000000407	-	18.25456	29.22456	7.284564	0.249262	-2.00427	0.030626	Ring finger protein 122 [Source:HGNCSymbol;Acc:HGNC:21147]
ENSOARG00000014842	COL6A5	360.5958	575.3434	145.8483	0.253498	-1.97995	0.000152	Collagen, type VI, alpha 5 [Source:HGNCSymbol;Acc:HGNC:26674]
ENSOARG00000015728	-	82.42573	131.1324	33.71905	0.257137	-1.95939	0.004437	Uncharacterized protein [Source:UniProtKB/TrEMBL;Acc:W5Q2E4]
ENSOARG00000001026	SLC13A5	18.44777	29.10817	7.78737	0.267532	-1.90222	0.039356	Solute carrier family 13 (sodium-dependent citratetransporter), member 5 [Source:HGNCSymbol;Acc:HGNC:23089]
ENSOARG00000008418	SOSTDC1	25.33127	39.75228	10.91027	0.274456	-1.86535	0.0099	Sclerostin domain containing 1 [Source:HGNCSymbol;Acc:HGNC:21748]
ENSOARG00000008702	-	59.7357	93.66627	25.80513	0.275501	-1.85987	0.007681	Uncharacterized protein [Source:UniProtKB/TrEMBL;Acc:W5PFX6]
ENSOARG00000017898	-	15.98647	25.0591	6.913837	0.275901	-1.85778	0.04882	Uncharacterized protein [Source:UniProtKB/TrEMBL;Acc:W5Q913]
ENSOARG00000009256	CD3G	96.12402	150.5393	41.70871	0.277062	-1.85172	0.030318	CD3g molecule, gamma (CD3-TCR complex) [Source:HGNCSymbol;Acc:HGNC:1675]
ENSOARG00000011756	HSPH1	1870.521	2922.127	818.9147	0.280246	-1.83523	0.027211	Heat shock 105kDa/110kDa protein 1 [Source:HGNCSymbol;Acc:HGNC:16969]
ENSOARG00000005501	SCN3A	56.6262	88.39153	24.86088	0.281259	-1.83003	0.013661	Sodium channel, voltage-gated, type III, alphasubunit [Source:HGNC Symbol;Acc:HGNC:10590]
ENSOARG00000000355	AGER	36.7374	57.33066	16.14414	0.281597	-1.8283	0.006165	Leucine rich repeat containing 31 [Source:HGNCSymbol;Acc:HGNC:26261]
ENSOARG00000013999	MSLN	19.12819	29.82901	8.427376	0.282523	-1.82356	0.028363	Mesothelin [Source:HGNC Symbol;Acc:HGNC:7371]
ENSOARG00000016200	CACNA1D	54.14378	84.3887	23.89886	0.2832	-1.82011	0.026233	Calcium channel, voltage-dependent, L type, alpha1D subunit [Source:HGNC Symbol;Acc:HGNC:1391]
ENSOARG00000019146	TGM1	21.34016	33.19118	9.489137	0.285893	-1.80645	0.036665	Transglutaminase 1 [Source:HGNCSymbol;Acc:HGNC:11777]
ENSOARG00000019502	CELSR1	336.0651	522.3256	149.8046	0.286803	-1.80187	6.98E-05	Cadherin, EGF LAG seven-pass G-type receptor 1 [Source:HGNC Symbol;Acc:HGNC:1850]
ENSOARG00000005117	THBD	650.6692	1007.258	294.0806	0.291962	-1.77615	0.037344	Thrombomodulin [Source:HGNC Symbol;Acc:HGNC:11784]
ENSOARG00000015517	PLCL2	463.3924	714.2877	212.4971	0.297495	-1.74906	0.026627	Phospholipase C-like 2 [Source:HGNCSymbol;Acc:HGNC:9064]
ENSOARG00000003077	AP3M2	22.39136	34.42006	10.36267	0.301065	-1.73185	0.040011	Adaptor-related protein complex 3, mu 2 subunit [Source:HGNC Symbol;Acc:HGNC:570]
ENSOARG00000004809	-	32.62922	50.10154	15.15691	0.302524	-1.72488	0.027949	Uncharacterized protein [Source:UniProtKB/TrEMBL;Acc:W5P401]
ENSOARG00000014276	SLC24A2	28.30474	43.08744	13.52203	0.313828	-1.67196	0.049885	Solute carrier family 24 (sodium/potassium/calciumexchanger), member 2 [Source:HGNC Symbol;Acc:HGNC:10976]
ENSOARG00000021049	-	23.39826	35.53159	11.26493	0.31704	-1.65726	0.035339	Uncharacterized protein [Source:UniProtKB/TrEMBL;Acc:W5QIS1]
ENSOARG00000013099	FBXL7	71.50825	108.2	34.81646	0.321779	-1.63586	0.00148	F-box and leucine-rich repeat protein 7 [Source:HGNC Symbol;Acc:HGNC:13604]

ENSOARG00000010841	DNAH11	88.091	133.2555	42.92646	0.322136	-1.63426	0.004555	Dynein, axonemal, heavy chain 11 [Source:HGNCSymbol;Acc:HGNC:2942]
ENSOARG00000020882	TEX9	57.87596	87.32628	28.42563	0.325511	-1.61922	0.034449	Testis expressed 9 [Source:HGNCSymbol;Acc:HGNC:29585]
ENSOARG00000017739	CAPN6	179.9035	270.7374	89.06952	0.328989	-1.60389	0.001041	Calpain 6 [Source:HGNC Symbol;Acc:HGNC:1483]
ENSOARG00000000581	LRRC31	43.29512	65.09673	21.49351	0.330178	-1.59868	0.008169	Advanced glycosylation end product-specificreceptor [Source:HGNC Symbol;Acc:HGNC:320]
ENSOARG00000002466	-	337.3099	506.7324	167.8875	0.331314	-1.59373	0.045742	-
ENSOARG00000021000	-	85.95493	128.5734	43.33645	0.337056	-1.56894	0.019898	Uncharacterized protein [Source:UniProtKB/TrEMBL;Acc:W5QIL5]
ENSOARG00000013163	ACSM3	38.58571	57.67949	19.49193	0.337935	-1.56518	0.016685	Acyl-CoA synthetase medium-chain family member 3 [Source:HGNC Symbol;Acc:HGNC:10522]
ENSOARG00000006333	PCBD1	71.7466	107.0324	36.46078	0.340652	-1.55363	0.014995	Pterin-4 alpha-carbinolaminedehydratase/dimerization cofactor of hepatocyte nuclearfactor 1 alpha [Source:HGNC Symbol;Acc:HGNC:8646]
ENSOARG00000009499	BST1	42.28088	63.02837	21.53338	0.341646	-1.54943	0.037646	Bone marrow stromal cell antigen 1 [Source:HGNCSymbol;Acc:HGNC:1118]
ENSOARG00000000317	LOX	42.35598	63.10416	21.60781	0.342415	-1.54618	0.046977	A kinase (PRKA) anchor protein 5 [Source:HGNCSymbol;Acc:HGNC:375]
ENSOARG00000019888	PTPN22	72.07532	107.1727	36.97795	0.345031	-1.5352	0.008623	Protein tyrosine phosphatase, non-receptor type 22(lymphoid) [Source:HGNC Symbol;Acc:HGNC:9652]
ENSOARG00000009653	RGS2	533.4747	791.654	275.2954	0.347747	-1.52389	1.46E-06	Regulator of G-protein signaling 2 [Source:HGNCSymbol;Acc:HGNC:9998]
ENSOARG00000008143	SMPDL3A	62.71123	92.55886	32.8636	0.355056	-1.49388	0.048429	Sphingomyelin phosphodiesterase, acid-like 3A [Source:HGNC Symbol;Acc:HGNC:17389]
ENSOARG00000013664	SKA3	34.28178	50.25835	18.30522	0.364222	-1.45711	0.031174	Spindle and kinetochore associated complex subunit3 [Source:HGNC Symbol;Acc:HGNC:20262]
ENSOARG00000006374	-	40.57881	59.31157	21.84605	0.368327	-1.44094	0.037691	-
ENSOARG00000007202	SPON1	143.31	209.4094	77.21048	0.368706	-1.43946	0.03278	Spondin 1, extracellular matrix protein [Source:HGNC Symbol;Acc:HGNC:11252]
ENSOARG00000008468	EMB	46.9727	68.41517	25.53022	0.373166	-1.42211	0.029456	Embigin [Source:HGNC Symbol;Acc:HGNC:30465]
ENSOARG00000006370	ASF1B	60.21145	87.36596	33.05695	0.378373	-1.40212	0.04953	Anti-silencing function 1B histone chaperone [Source:HGNC Symbol;Acc:HGNC:20996]
ENSOARG00000014749	ALDH8A1	31.62599	45.87795	17.37403	0.378701	-1.40087	0.040165	Aldehyde dehydrogenase 8 family, member A1 [Source:HGNC Symbol;Acc:HGNC:15471]
ENSOARG00000006007	NGFR	29.25298	42.3564	16.14956	0.381278	-1.39109	0.046984	Nerve growth factor receptor [Source:HGNCSymbol;Acc:HGNC:7809]
ENSOARG00000010184	AK4	66.23334	94.9576	37.50908	0.395009	-1.34004	0.046676	Adenylate kinase 4 [Source:HGNCSymbol;Acc:HGNC:363]
ENSOARG00000018772	ACAD10	2693.832	3843.829	1543.834	0.40164	-1.31603	2.96E-06	Uncharacterized protein [Source:UniProtKB/TrEMBL;Acc:W5QBS0]
ENSOARG00000000991	AKAP5	128.8338	183.7849	73.88278	0.402007	-1.31471	0.000907	Lysyl oxidase [Source:HGNC Symbol;Acc:HGNC:6664]
ENSOARG00000001534	PIEZO2	180.3137	256.659	103.9684	0.405084	-1.30371	0.001669	Piezo-type mechanosensitive ion channel component 2 [Source:HGNC Symbol;Acc:HGNC:26270]
ENSOARG00000011453	SELENOW	982.3727	1382.871	581.8741	0.420772	-1.24889	1.78E-05	Selenoprotein W, 1 [Source:HGNCSymbol;Acc:HGNC:10752]
ENSOARG00000020413	SERPINE2	252.0039	354.3999	149.608	0.422144	-1.24419	0.034656	Serpin peptidase inhibitor, clade E (nexin,plasminogen activator inhibitor type 1), member 2 [Source:HGNC Symbol;Acc:HGNC:8951]

ENSOARG00000006895	PODXL2	58.03811	81.61601	34.4602	0.422224	-1.24392	0.040804	Podocalyxin-like 2 [Source:HGNCSymbol;Acc:HGNC:17936]
ENSOARG00000015414	-	70.05157	98.34179	41.76134	0.424655	-1.23564	0.043128	Uncharacterized protein [Source:UniProtKB/TrEMBL;Acc:W5Q1E8]
ENSOARG00000009911	PROM1	74.83146	104.8975	44.76542	0.426754	-1.22852	0.012325	Prominin 1 [Source:HGNC Symbol;Acc:HGNC:9454]
ENSOARG00000004886	UBXN11	68.44502	95.66243	41.2276	0.43097	-1.21434	0.04365	UBX domain protein 11 [Source:HGNCSymbol;Acc:HGNC:30600]
ENSOARG00000020279	AHSA2	124.7293	174.0873	75.37141	0.432952	-1.20772	0.01114	AHA1, activator of heat shock 90kDa protein ATPasehomolog 2 (yeast) [Source:HGNC Symbol;Acc:HGNC:20437]
ENSOARG00000011513	IRF8	155.9224	217.3393	94.50547	0.434829	-1.20148	0.038289	Interferon regulatory factor 8 [Source:HGNCSymbol;Acc:HGNC:5358]
ENSOARG00000006809	-	67.60541	94.2091	41.00171	0.43522	-1.20018	0.024833	Uncharacterized protein [Source:UniProtKB/TrEMBL;Acc:W5PA32]
ENSOARG00000002973	KCNN3	67.73298	94.32877	41.1372	0.436105	-1.19725	0.02145	Potassium intermediate/small conductance calcium-activated channel, subfamily N, member 3 [Source:HGNCSymbol;Acc:HGNC:6292]
ENSOARG00000011945	ELN	319.0865	442.2779	195.8951	0.442923	-1.17487	0.010825	Elastin [Source:HGNC Symbol;Acc:HGNC:3327]
ENSOARG00000011841	ANPEP	1945.271	2691.119	1199.424	0.445697	-1.16586	0.004394	Alanyl (membrane) aminopeptidase [Source:HGNCSymbol;Acc:HGNC:500]
ENSOARG00000017314	GALNT6	59.55921	82.34306	36.77536	0.446612	-1.16291	0.044191	Polypeptide N-acetylgalactosaminyltransferase 6 [Source:HGNC Symbol;Acc:HGNC:4128]
ENSOARG00000017084	-	331.4105	457.2848	205.5362	0.449471	-1.1537	0.000658	Uncharacterized protein [Source:UniProtKB/TrEMBL;Acc:W5Q6J5]
ENSOARG00000021170	ARG2	469.6509	647.6833	291.6184	0.450248	-1.15121	0.000824	Uncharacterized protein [Source:UniProtKB/TrEMBL;Acc:W5QJ54]
ENSOARG00000004796	CD22	205.2136	282.9537	127.4735	0.45051	-1.15037	0.032273	CD22 molecule [Source:HGNC Symbol;Acc:HGNC:1643]
ENSOARG00000016110	BRIP1	71.06383	97.9214	44.20627	0.451446	-1.14737	0.017653	BRCA1 interacting protein C-terminal helicase 1 [Source:HGNC Symbol;Acc:HGNC:20473]
ENSOARG00000002340	ZGRF1	39.52139	54.41874	24.62404	0.452492	-1.14404	0.048402	Zinc finger, GRF-type containing 1 [Source:HGNCSymbol;Acc:HGNC:25654]
ENSOARG00000015400	WNT5A	249.4285	343.3657	155.4914	0.452845	-1.14291	0.000791	Wingless-type MMTV integration site family, member5A [Source:HGNC Symbol;Acc:HGNC:12784]
ENSOARG00000014093	LRRN1	87.97216	121.0479	54.89638	0.453509	-1.1408	0.013215	Leucine rich repeat neuronal 1 [Source:HGNCSymbol;Acc:HGNC:20980]
ENSOARG00000020170	FAM161A	77.045	105.9777	48.1123	0.453985	-1.13928	0.014715	Family with sequence similarity 161, member A [Source:HGNC Symbol;Acc:HGNC:25808]
ENSOARG00000008262	SCN2B	65.00697	89.38859	40.62535	0.45448	-1.13771	0.01912	Sodium channel, voltage-gated, type II, betasubunit [Source:HGNC Symbol;Acc:HGNC:10589]
ENSOARG00000008351	SLCO2A1	185.0614	252.8079	117.3148	0.464047	-1.10766	0.001591	Solute carrier organic anion transporter family,member 2A1 [Source:HGNC Symbol;Acc:HGNC:10955]
ENSOARG00000001884	RIMKLB	71.57948	97.66675	45.49221	0.46579	-1.10225	0.025082	Ribosomal modification protein rimK-like familymember B [Source:HGNC Symbol;Acc:HGNC:29228]
ENSOARG00000020996	ATP8B4	381.9666	520.9764	242.9568	0.466349	-1.10052	0.034074	ATPase, class I, type 8B, member 4 [Source:HGNCSymbol;Acc:HGNC:13536]
ENSOARG00000018978	RNASEL	90.24986	122.9794	57.52029	0.467723	-1.09627	0.017866	Ribonuclease L (2',5'-oligoisoadenylate synthetase-dependent) [Source:HGNC Symbol;Acc:HGNC:10050]
ENSOARG00000020768	-	327.0656	443.6843	210.4469	0.474317	-1.07608	0.036012	Uncharacterized protein [Source:UniProtKB/TrEMBL;Acc:W5QHX2]
ENSOARG00000017961	PTPLAD1	121.3198	164.1246	78.51501	0.478387	-1.06375	0.013467	Protein tyrosine phosphatase-like A domaincontaining 1 [Source:HGNC

								Symbol;Acc:HGNC:24175]
ENSOARG00000018575	ZKSCAN2	66.63701	90.10219	43.17183	0.479143	-1.06147	0.028274	Zinc finger with KRAB and SCAN domains 2 [Source:HGNC Symbol;Acc:HGNC:25677]
ENSOARG00000008878	CD84	215.2776	290.9685	139.5868	0.479732	-1.0597	0.049752	CD84 molecule [Source:HGNC Symbol;Acc:HGNC:1704]"
ENSOARG00000006056	LPAR4	80.03908	107.9253	52.15284	0.483231	-1.04922	0.03311	Lysophosphatidic acid receptor 4 [Source:HGNCSymbol;Acc:HGNC:4478]
ENSOARG00000015273	BORA	99.32588	133.8093	64.84241	0.484588	-1.04517	0.011391	Bora, aurora kinase A activator [Source:HGNCSymbol;Acc:HGNC:24724]
ENSOARG00000012752	-	55.40205	74.60768	36.19642	0.485157	-1.04348	0.044335	Uncharacterized protein [Source:UniProtKB/TrEMBL;Acc:W5PT72]
ENSOARG00000019689	MANSC4	192.0284	258.4676	125.5892	0.485899	-1.04127	0.015716	MANSC domain containing 4 [Source:HGNCSymbol;Acc:HGNC:40023]
ENSOARG00000012355	WEE1	184.4189	247.869	120.9688	0.488035	-1.03494	0.005429	WEE1 G2 checkpoint kinase [Source:HGNCSymbol;Acc:HGNC:12761]
ENSOARG00000005180	HLTF	613.6813	823.326	404.0366	0.490737	-1.02698	0.005168	Helicase-like transcription factor [Source:HGNCSymbol;Acc:HGNC:11099]
ENSOARG00000010529	KAZN	87.91252	117.6481	58.17697	0.4945	-1.01596	0.024627	Kazrin, periplakin interacting protein [Source:HGNCSymbol;Acc:HGNC:29173]
ENSOARG00000013977	SSX2IP	65.89572	88.07162	43.71983	0.496412	-1.01039	0.045396	Synovial sarcoma, X breakpoint 2 interactingprotein [Source:HGNC Symbol;Acc:HGNC:16509]
ENSOARG00000016590	TRIM29	455.6445	607.9072	303.3819	0.49906	-1.00272	0.014652	Tripartite motif containing 29 [Source:HGNCSymbol;Acc:HGNC:17274]
ENSOARG00000018430	-	92.9954	124.0385	61.95233	0.499461	-1.00156	0.023468	-
